# Regenerated Viscose Fibers Enabled by Recycled Cotton Pulps with Different Degrees of Polymerization from Waste Textiles

**DOI:** 10.3390/polym18111302

**Published:** 2026-05-26

**Authors:** Huansheng Cai, Lin Chen, Xiuli Wang

**Affiliations:** The Collaborative Innovation Center for Eco-Friendly and Fire-Safety Polymeric Materials (MoE), National Engineering Laboratory of Eco-Friendly Polymeric Materials (Sichuan), State Key Laboratory of Advanced Polymer Materials, College of Chemistry, Sichuan University, Chengdu 610064, China; 2023222030153@stu.scu.edu.cn

**Keywords:** recycling of waste fabrics, recycled cotton pulp, degree of polymerization, viscose fiber, mechanical properties

## Abstract

High-value recycling of waste cotton-containing fabrics is crucial for the sustainable development of the textile industry. In this study, cotton pulps with different degrees of polymerization (DP = 512–789) from waste polyester/cotton fabrics are systematically evaluated for viscose fiber production. The insolubles in the spinning solution and the effects of DP on its rheological behavior are examined. Based on the mechanical properties of the prepared viscose fibers, the spinning parameters (draw ratio, coagulation bath temperature, and H_2_SO_4_ concentration) are optimized. The results show that recycled pulps can produce spinning solutions without insolubles, indicating good spinnability and viscoelastic behavior similar to commercial wood pulp. Higher DP increases apparent viscosity and high-frequency elasticity. Under the optimal spinning conditions (draw ratio 1:1.13, coagulation bath temperature 40 °C, and H_2_SO_4_ concentration 8%), the viscose fibers prepared from recycled cotton pulp with DP = 789 achieve a dry tenacity of 2.31 cN/dtex, which is 37.5% higher than that of wood pulp-based viscose fibers, and exhibit higher elongation at break. This study provides a basis for quality control and process improvement in producing high-tenacity viscose fibers from recycled cotton pulp, paving the way for high-value recycling of waste cotton-containing fabrics.

## 1. Introduction

The annual global consumption of fibrous materials has exceeded 92 million tons and is projected to reach 160 million tons by 2050 [1], generating substantial textile waste that imposes significant environmental and resource challenges. Textile waste is mainly managed through landfilling and incineration, which releases non-degradable microfibers and greenhouse gases while causing irreversible loss of polymeric resources [2,3,4,5]. Mechanical recycling is simple and low-cost, but severely degrades fiber properties, restricting its use to low-value products [6,7]. Moreover, the complex multi-component blended structures of waste textiles, along with impurities, such as dyes and additives, and the presence of metal and plastic accessories, significantly complicate their recycling. Currently, less than 1% of textile waste is reused at the same quality [4], highlighting the urgent need for high-value recycling strategies.

Cotton is the second most widely used textile fiber after polyester, accounting for approximately 24% of textile waste [8]. Currently, waste cotton is mainly mechanically recycled into nonwoven materials with relatively simple processing routes and broad industrial demand. However, cotton cultivation and processing are associated with intensive pesticide use and greenhouse gas emissions, imposing notable environmental burdens [9]. Regenerated cellulose fibers, such as viscose and Lyocell, possess chemical structures similar to natural cotton but offer superior uniformity, hygroscopicity, and designability [10,11,12,13,14,15]. Recovering cellulose from waste cotton textiles through chemical methods and converting it into regenerated viscose fibers represents an effective route for high-value utilization. Among these, Lyocell fibers, produced via the *N*-methylmorpholine *N*-oxide (NMMO) process, have emerged as an environmentally friendly and rapidly developing route for regenerated cellulose fiber production, yielding fibers with excellent mechanical properties and process stability. Several studies have explored Lyocell production from both virgin and waste cellulose sources, demonstrating its potential for sustainable fiber manufacturing [16,17,18]. Viscose fiber production is a traditional and well-established method for regenerated cellulose fibers, involving chemical treatment of cellulose through xanthation and regeneration [19,20,21]. In comparison, the industrial capacity of Lyocell fibers remains limited, and their share in the regenerated cellulose fibers market is relatively low [22,23], making it difficult to fully digest large volumes of waste cotton textiles.

Cotton fibers are often blended with synthetic fibers such as polyester [1,24,25], and the presence of non-cotton components can severely interfere with the spinning of regenerated viscose fibers. Therefore, efficient separation of cotton from non-cotton components is critical for high-value utilization. Chemical recycling of cotton-containing textiles typically relies on acid or alkali treatments to achieve the separation of various components. Acid hydrolysis (usually with hydrochloric or phosphoric acid) can readily cleave glycosidic bonds in cotton fibers by protonation, converting cotton into small-molecular sugars or furfural [26,27,28], which cannot be regenerated into fibers. Alkali hydrolysis (such as sodium or potassium hydroxide) targets the carbonyl groups of polyester via nucleophilic attack of OH^−^, enabling hydrolysis of polyester and separation from cotton [29,30,31], yielding cotton pulps with relatively high purity. However, existing studies have focused on the separation of cotton and non-cotton components. Systematic investigations into the downstream high-value utilization of recycled cotton pulps are still limited. In particular, the processability of recycled cotton pulps and the resulting impact on viscose fiber properties remain poorly understood. Even in industrial-scale chemical purification of waste cotton textiles using alkaline aqueous systems, the recycled cotton pulps require incorporation with more than 50% virgin wood pulp to achieve spinnability [32], which limits their potential in high-performance regenerated viscose fiber production. Dissolving-grade cellulose for viscose fiber production must meet specific quality parameters [33,34,35], including high α-cellulose content (preferably >92%) and an appropriate degree of polymerization (DP) (for cotton pulp, typically 500–800). These requirements are precisely why recycled cotton pulps need to be blended with a large proportion of virgin wood pulp. Among these parameters, DP is particularly critical, as it directly affects the spinning process and the mechanical properties of regenerated viscose fibers. Therefore, investigating the spinning process of recycled cotton pulps and the effects of the cotton pulps’ DP on the mechanical properties of regenerated viscose fibers is crucial for achieving high-value utilization of waste cotton fibers.

**Figure 1:**
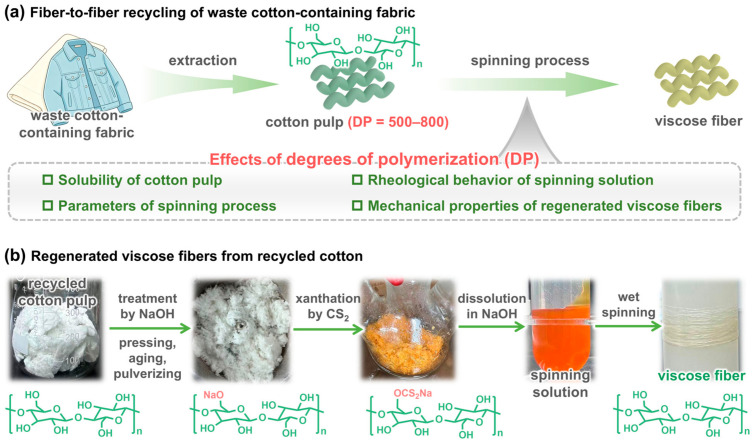
(**a**) Schematic of fiber-to-fiber recycling from waste cotton-containing fabrics to regenerated viscose fibers, illustrating the effects of DP of recycled cotton pulp on the viscose production process. (**b**) Process flow for producing regenerated viscose fibers from recycled cotton pulp.

Our group has developed a novel process that can prepare regenerated viscose fibers by 100% recycled cotton from waste cotton-containing textiles, thereby enabling a closed-loop recycling from waste cotton fibers to regenerated viscose fibers (Figure 1a). This process efficiently removes dyes and synthetic fibers (polyester fiber) to obtain high-quality cotton pulp with DP in the range of 500–800. After alkalization and xanthation, the recycled cotton pulp is converted into a spinning solution and wet-spun into regenerated viscose fibers (Figure 1b), with its DP strongly influencing solution behavior and fiber mechanical properties. This study aims to systematically investigate the influence of the DP of recycled cotton pulp on the spinning process and the properties of regenerated viscose fibers. It is expected that controlling the DP of recycled cotton pulp can significantly affect the rheological behavior of spinning solutions and the mechanical performance of the resulting viscose fibers. To this end, we systematically evaluate the insoluble content and rheological behavior of spinning solutions, optimize spinning process parameters, and investigate the mechanical performance of regenerated viscose fiber. The findings provide guidance for the high-value utilization of waste cotton fibers and pave the way for more sustainable development in the textile industry.

## 2. Materials and Methods

### 2.1. Materials

Post-consumer polyester/cotton blends (70% cotton and 30% polyester) derived from used denim workwear and commercial wood pulp were supplied by Tangshan Sanyou Company, Tangshan, China. Sodium hydroxide (NaOH) and carbon disulfide (CS_2_) were purchased from Aladdin (Shanghai, China). Sodium sulfate (Na_2_SO_4_) and zinc sulfate heptahydrate (ZnSO_4_·7H_2_O) were purchased from Shanghai Adamas Reagent Co., Ltd., Shanghai, China. Concentrated sulfuric acid (H_2_SO_4_) was from Chengdu Kelong Chemical Reagent Factory, Chengdu, China. All the above reagents were analytically pure and were not further purified before use.

### 2.2. Preparation of Recycled Cotton Pulp

Waste polyester/cotton fabrics were used as the raw material, and the detailed preparation procedure of the recycled cotton pulp was performed as described in reference [36]. By controlling the reaction conditions, four recycled cotton pulps with different DP were obtained, with average DP of 789, 750, 695, and 512, respectively. The recycled cotton pulps of different DP exhibited high α-cellulose contents of 95–97%.

### 2.3. Preparation of Viscose Spinning Solution and Wet Spinning of Fibers

Firstly, the recycled cotton pulp was pretreated with 280 g/L NaOH solution (solid-to-liquid ratio of 1:25) at 50 °C for 20 min. After squeezing, the pulp was subjected to a second alkali treatment with 14 wt% NaOH, followed by pressing to a moisture content of 60–80%. Subsequently, the pulp was sealed and aged at 40 °C for 6 h. After aging, the pulp was mechanically disintegrated and reacted with carbon disulfide (40% by mass of the dry pulp) under a nitrogen atmosphere at 29 °C until a deep orange color developed (approximately 1.5 h). Finally, an 8 wt% NaOH solution was added to adjust the solid content of the spinning solution to 8 wt%, and the mixture was stirred until a clear spinning solution was obtained. Residual bubbles were removed by centrifuging at 6000 rpm for 5 min.

The spinning solution was introduced into the wet-pinning machine, with 6–9% H_2_SO_4_, 12 wt% ZnSO_4_, and 14 wt% Na_2_SO_4_ employed as the coagulation bath and maintained at 20–50 °C. Spinning was conducted using a 20 × 0.10 mm spinneret. The draw ratio was adjusted via extrusion and winding speeds. The resulting filaments were collected, washed with distilled water to remove residual salts, and dried at low temperature.

### 2.4. Characterizations

The DP of recycled cotton pulp was determined according to ISO 5351:2010 “Pulps—Determination of limiting viscosity number in cupri-ethylenediamine (CED) solution” [37] using a capillary viscometer. All viscosity measurements were performed at 25.0 ± 0.1 °C in accordance with the standard. DP was calculated using the empirical Mark–Houwink relation commonly adopted for cellulose in CED solution:

DP^0.905^ = 0.75[*η*] (1)

where the intrinsic viscosity [*η*] is obtained from the measured flow time and expressed in mL/g, and 0.75 is an empirical constant for cellulose in 0.5 mol/L CED solution.

FTIR spectra were recorded on a Nicolet 6700 spectrophotometer (Thermo Fisher Scientific, Waltham, MA, USA) scanning from 4000 cm^−1^ to 500 cm^−1^. The sample was tableted with potassium bromide in a mass ratio of 1:100.

The spinning solution prepared from recycled cotton pulp was observed using a Smartzoom 5 digital microscope (Carl Zeiss Microscopy GmbH, Oberkochen, Germany).

The rheological behavior of the viscose spinning solutions was measured using a Discovery HR-2 rotational rheometer (TA Instruments, New Castle, DE, USA) with a 40 mm parallel-plate geometry and 1000 μm gap at 25 °C. Steady shear tests were performed with a shear rate of 0.1–100 s^−1^. Dynamic oscillatory measurements were conducted within the linear viscoelastic region over an angular frequency range of 0.2–628 rad s^−1^, and the storage modulus and loss modulus were recorded. To obtain the zero-shear viscosity and characteristic relaxation time for quantitative evaluation of molecular chain entanglement, the complex viscosity derived from dynamic frequency sweep data was further fitted by the Carreau–Yasuda model:

(2) $$\eta^{*}\left(\omega \right)=\eta_{\infty }+\left(\eta_{0}-\eta_{\infty }\right){\left[1+{\left(\lambda \omega \right)}^{a}\right]}^{\frac{n-1}{a}}$$

where η^*^(*ω*) is the complex viscosity at angular frequency *ω*, *η*_0_ and *η*_∞_ are the zero-shear and infinite-shear viscosities, respectively, *λ* is the relaxation time, *n* is the power-law index describing the shear-thinning behavior, and *a* is the Yasuda parameter controlling the width of the transition region between the Newtonian plateau and the shear-thinning regime.

The mechanical properties of the viscose fibers were evaluated using an electronic single-fiber strength tester (LLY-06B, Laizhou Electronic Instrument Co., Ltd., Laizhou, China) to measure the dry-state tensile strength and elongation at break of the fibers. At least 20 filaments were tested for each sample, then a small number of anomalous data points were excluded, and 20 valid replicate data points were ultimately retained for statistical analysis.

The micromorphology of the viscose fibers was observed on scanning electron microscopy (JSE-7500F, JEOL Ltd., Tokyo, Japan) under the condition of 15 kV, and the samples were sprayed with gold before the test.

## 3. Results and Discussion

### 3.1. Presence of Insolubles in Viscose Spinning Solutions

High molecular weight, long-chain cellulose in cotton pulp exhibits high crystallinity and low reactivity, which often leads to incomplete xanthation. The fraction not converted into cellulose xanthate remains as insoluble particulate content in the spinning solution. Additionally, incompletely depolymerized polyester can persist as fibrous residues. This insoluble content may cause spinneret clogging, instability during the spinning process, and deterioration of the mechanical properties of regenerated viscose fibers. Therefore, the presence of insolubles in the spinning solution is a critical indicator for assessing the spinnability of the pulp and is closely related to the DP of the pulp.

**Figure 2:**
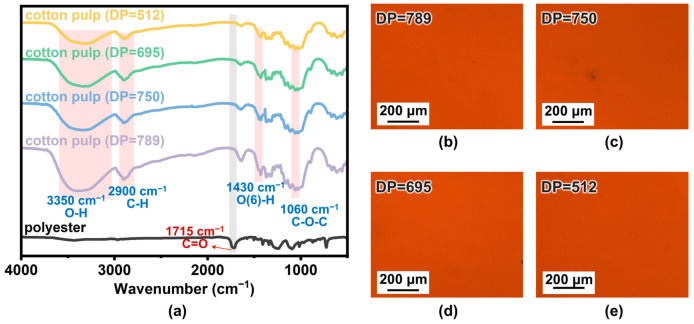
FTIR spectra of polyester fibers and recycled cotton pulps with DP values of 789, 750, 695, and 512 (**a**), and microscope images of spinning solutions prepared from recycled cotton pulps with DP values of 789 (**b**), 750 (**c**), 695 (**d**), and 512 (**e**).

As shown in Figure 2a, the characteristic carbonyl absorption peak of polyester completely disappears in the FTIR spectra of all recycled cotton pulps, indicating the absence of polyester residues. In addition, the FTIR spectra of all recycled cotton pulps exhibit the characteristic absorption bands of cellulose. The broad band at approximately 3350 cm^−1^ and the peak at 2900 cm^−1^ are assigned to the O-H and C-H stretching vibrations of cellulose, respectively. The absorption band at 1430 cm^−1^ corresponds to the characteristic bending vibration of O(6)-H, while the strong peak at 1060 cm^−1^ is attributed to the C-O-C stretching vibration of the β-1,4-glycosidic backbone. Microscopic images of spinning solutions prepared from recycled cotton pulps with different DP are shown in Figure 2b–e. The solid content of all spinning solutions, corresponding to the cellulose xanthate content, was 8 wt%. No obvious insoluble particles or impurities were observed within the field of view. The spinning solutions exhibit a uniform and continuous morphology, providing a solid foundation for stable spinning. These observations suggest that, within the investigated DP range, the overall reactivity of the cellulose chains was sufficient to enable complete xanthation and subsequent dissolution in the alkaline solution. Moreover, the high purity of the recycled cotton pulps indirectly validates the effectiveness of the developed process in removing polyester residues.

### 3.2. Rheological Behavior of Spinning Solutions

The absence of insoluble content is a prerequisite for stable spinning, while rheological properties directly determine both the stability of the spinning process and the mechanical properties of regenerated fibers. To this end, steady-state and dynamic rheological tests were conducted to systematically investigate the influence of the DP of recycled cotton pulps on the rheological behavior of viscose spinning solutions, with commercial wood pulp (DP = 455) as a reference to evaluate their suitability for the spinning process of viscose fiber. To ensure a reliable comparison, the solid content of both recycled cotton pulp-derived and wood pulp-derived spinning solutions was 8 wt%, and all rheological measurements were carried out at 25 °C.

**Figure 3:**
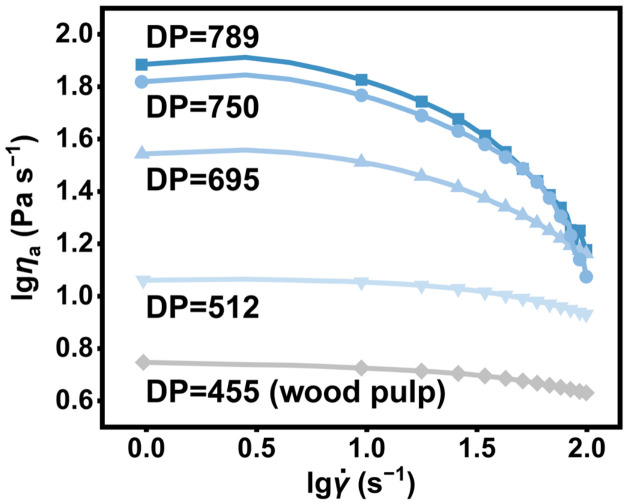
Steady shear curves of spinning solutions prepared from recycled cotton pulps with different DP and commercial wood pulp. *η*_a_ on the vertical axis represents the apparent viscosity of the spinning solutions.

Figure 3 presents the steady-state flow curves of spinning solutions. All solutions exhibit typical shear-thinning behavior, where the apparent viscosity (*η*_a_) decreases with increasing shear rate ($\dot{\gamma }$). This behavior facilitates smooth extrusion of the spinning solution through the spinneret under high-shear conditions and helps maintain shape stability in the low-shear post-extrusion region. Notably, at very low shear rates, spinning solutions prepared from high-DP recycled cotton pulps (DP = 789, 750, and 695) exhibit a slight transient increase in apparent viscosity, attributable to the elastic response and transient entanglement of cellulose chains, whereas spinning solutions from lower-DP samples do not show this behavior [38,39]. Moreover, increasing the DP of the recycled cotton pulp resulted in a significant increase in the apparent viscosity of the spinning solutions, while the critical shear rate shifted toward lower values. This behavior is attributed to the higher entanglement density associated with longer cellulose molecular chains. The enhanced chain entanglement increases the zero-shear viscosity and promotes earlier disruption of the entanglement network under shear.

Compared with commercial wood pulp (DP = 455), spinning solutions prepared from recycled cotton pulps exhibited consistently higher apparent viscosities. This moderately increased viscosity is beneficial for maintaining flow stability during spinning. In addition, it facilitates molecular chain alignment along the flow direction during drawing, thereby contributing to the improved mechanical properties of the regenerated viscose fibers.

**Figure 4:**
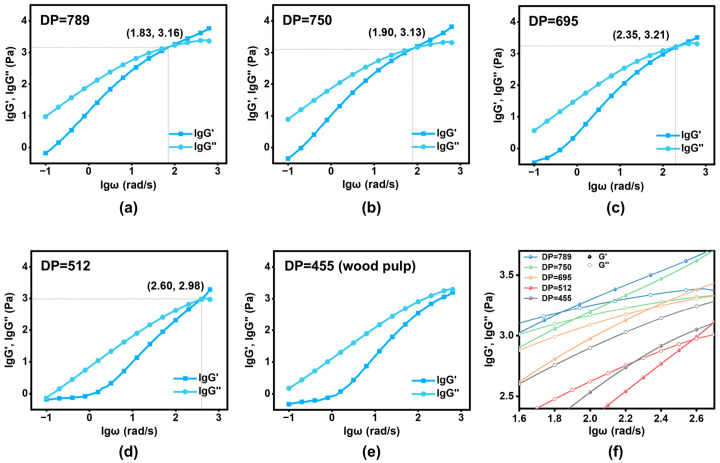
Dynamic rheological curves of spinning solutions prepared from recycled cotton pulps with DP values of 789 (**a**), 750 (**b**), 695 (**c**), 512 (**d**), commercial wood pulp (**e**), and a comparative overlay of all samples (**f**).

Figure 4 shows the dynamic rheological curves of the spinning solutions. Both recycled cotton pulp and wood pulp solutions exhibit similar viscoelastic behavior. At low frequencies, the loss modulus (*G*″) exceeds the storage modulus (*G*′), indicating a predominantly viscous response and good flowability. With increasing frequency, *G*′ gradually surpasses *G*″, indicating a transition to an elasticity-dominated regime. This viscoelastic transition is favorable for molecular chain alignment during the spinning–drawing process and thereby reduces the risk of fiber breakage. Further analysis demonstrates that the DP of the pulp has a significant influence on the dynamic rheological properties. As DP increases, the viscous response at low frequencies becomes more pronounced, and the elasticity at high frequencies is enhanced. Figure 4f compares the critical crossover angular frequencies of all spinning solutions, which decrease progressively with increasing pulp DP. Although the wood pulp (DP = 455) exhibits higher *G*′ and *G*″ values than the recycled cotton pulp with DP = 512, these differences are mainly attributed to variations in raw material structure and solution homogeneity, whereas the crossover angular frequency is primarily governed by pulp DP. These observations reflect a higher entanglement density and longer chain relaxation times in spinning solutions prepared from higher-DP pulps.

To further quantitatively characterize the molecular chain entanglement density and relaxation behavior, the complex viscosity derived from dynamic rheological data was fitted by the Carreau–Yasuda model. The fitted zero-shear viscosity (*η*_0_) and characteristic relaxation time (*λ*), along with the power-law index (*n*) and transition parameter (*a*), are summarized in Table 1. All fittings yielded R^2^ > 0.999, indicating excellent agreement with the experimental data. As shown in Table 1, higher DP generally corresponds to larger *η*_0_ and *λ*, supporting that higher-DP cellulose molecular chains form a more stable and denser entanglement network with slower chain relaxation dynamics. A minor deviation in *λ* was observed between the DP = 789 and DP = 750 samples, which is likely due to fitting uncertainties, but the overall increasing trend with DP is clear. Consequently, higher-DP cotton pulps provide stronger structural support during drawing and promote molecular chain orientation, thereby enhancing the mechanical performance of the viscose fibers. Importantly, compared with commercial wood pulp solutions, spinning solutions prepared from recycled cotton pulps exhibit lower critical angular frequencies and higher *G*′ values at high frequencies (Figure 4), indicating a stronger elastic response during drawing. Table 1 further reveals that spinning solutions from recycled cotton pulps with higher DP exhibit larger zero-shear viscosity and relaxation time compared with those from commercial wood pulp. These rheological characteristics establish a favorable rheological foundation for the production of high-performance regenerated viscose fibers.

It is noteworthy that most rheological studies on viscose spinning solutions focus on steady-state flow, while dynamic viscoelastic properties remain underexplored [40,41,42]. Nevertheless, understanding the viscoelastic behavior of the spinning solution is essential because solution elasticity directly affects filament formation. In this work, we extend rheological characterization to dynamic measurements, linking pulp DP, solution behavior, and spinnability. From a viscoelastic perspective, stable spinning requires a balance between flowability at low frequencies and structural support at higher frequencies. Regarding the *G*′/*G*″ crossover, our viscose spinning solutions exhibit crossover angular frequencies in the range of 60–500 rad/s, which is consistent with the rheological behavior of low-concentration cellulose derivative solutions reported in the literature [43]. Within this intermediate crossover regime, a balance between chain entanglement and flowability is achieved, ensuring stable spinning. In addition, the DP = 789 sample exhibits the most favorable rheological balance for filament formation.

In summary, viscose spinning solutions prepared from recycled cotton pulps with different DP exhibit shear-thinning and viscoelastic transition behavior consistent with commercial wood pulp, confirming good spinnability. Furthermore, increasing DP endows the spinning solutions with higher apparent viscosity and stronger high-frequency elastic response, offering potential advantages during drawing orientation and providing experimental guidance for systematically optimizing DP to balance spinnability and fiber performance.

### 3.3. Optimization of Viscose Spinning Parameters

Dry-state tensile strength and elongation at break are crucial mechanical properties of viscose fibers, directly affecting their processing and end-use performance. Rheological analysis in Section 3.2 shows that higher-DP cotton pulps produce spinning solutions with higher apparent viscosity and stronger high-frequency elasticity. Therefore, spinning experiments using high-DP recycled cotton pulp (DP = 789) were conducted to examine the effects of draw ratio, coagulation bath temperature, and sulfuric acid concentration on tensile strength and elongation of regenerated viscose fibers, aiming to determine optimal spinning conditions. Notably, ZnSO_4_ and Na_2_SO_4_ concentrations were kept constant at conventional viscose spinning conditions, as their primary roles are to regulate coagulation kinetics and bath stability [44], whereas H_2_SO_4_ concentration plays a more dominant role in cellulose regeneration and fiber structure development.

**Figure 5:**
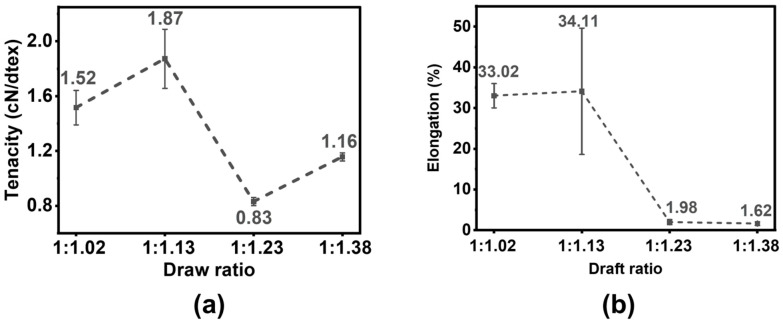
Effects of draw ratio on dry tenacity (**a**) and elongation at break (**b**) of regenerated viscose fibers. Coagulation bath temperature and H_2_SO_4_ concentration were 40 °C and 9%.

The effects of draw ratio on the mechanical properties of regenerated viscose fibers are shown in Figure 5. The coagulation bath temperature and H_2_SO_4_ concentration were kept constant at 40 °C and 9%, respectively. As the draw ratio increases, the dry-state tensile strength initially increases, reaching a maximum value of 1.87 cN/dtex at a draw ratio of 1:1.13. Conversely, further increases in the draw ratio lead to a reduction in strength (Figure 5a). A similar trend is also observed for elongation at break (Figure 5b). This behavior suggests that moderate drawing effectively promotes the axial alignment of cellulose molecular chains, leading to improved chain packing, increased structural regularity, and enhanced effective crystallinity. However, excessive drawing may induce localized stress concentration and structural imperfections within the fiber, thereby disrupting the continuity of the molecular network and resulting in deteriorated mechanical performance.

**Figure 6:**
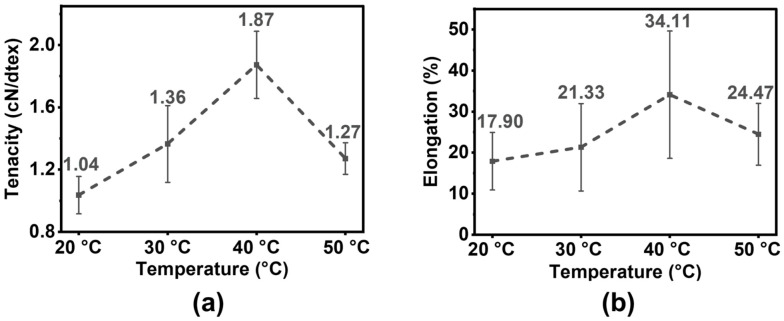
Effects of coagulation-bath temperature on dry tenacity (**a**) and elongation at break (**b**) of regenerated viscose fibers. The draw ratio and H_2_SO_4_ concentration were 1:1.13 and 9%.

The influence of coagulation bath temperature on the mechanical properties of regenerated viscose fibers is shown in Figure 6. Based on the previously determined optimal draw ratio (1:1.13), all subsequent experiments were carried out at this draw ratio and an H_2_SO_4_ concentration of 9% to ensure consistent comparison. As the coagulation bath temperature increases from 20 °C to 40 °C, the dry-state tensile strength significantly increases from 1.04 to 1.87 cN/dtex (Figure 6a), accompanied by a concurrent improvement in elongation at break (Figure 6b). This enhancement can be attributed to more balanced kinetics between cellulose xanthate decomposition and cellulose regeneration during the spinning process, which facilitates the formation of a more homogeneous fiber structure. However, further increasing the temperature to 50 °C leads to a simultaneous decline in both tensile strength and elongation at break. This deterioration suggests that excessively high temperatures accelerate the coagulation process, resulting in non-uniform coagulation and structural defects within the viscose fiber, thereby compromising fiber performance. Therefore, 40 °C provides an optimal balance between regeneration kinetics and structural integrity, yielding viscose fibers with improved mechanical performance and uniform morphology.

**Figure 7:**
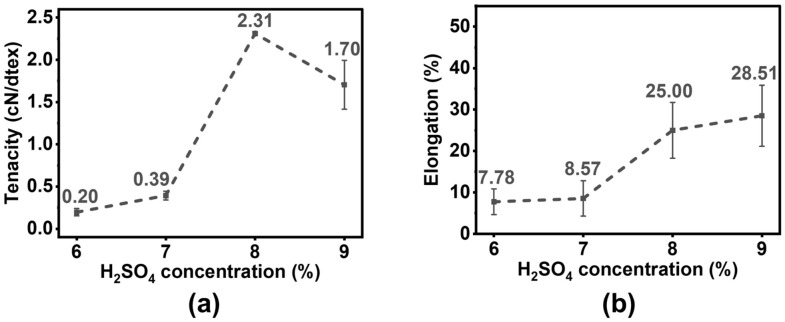
Effects of H_2_SO_4_ concentration on dry tenacity (**a**) and elongation at break (**b**) of regenerated viscose fibers. Draw ratio and coagulation bath temperature were 1:1.13 and 40 °C.

The effect of H_2_SO_4_ concentration on the mechanical properties of regenerated viscose fibers is shown in Figure 7. Increasing the acid concentration from 6% to 8% results in a marked improvement in dry-state tensile strength, which increases from 0.20 to 2.31 cN/dtex (Figure 7a). Meanwhile, the elongation at break also increases, indicating a simultaneous enhancement in fiber strength and ductility (Figure 7b). Further increasing the H_2_SO_4_ concentration to 9% leads to a reduction in tensile strength (1.70 cN/dtex), while the increase in elongation becomes less pronounced. This trend suggests that moderate acid concentration promotes an appropriate balance between cellulose xanthate decomposition and coagulation of viscose fibers, facilitating molecular chain orientation and the formation of a more compact fiber structure. In contrast, excessively high acid concentration accelerates the coagulation process, leading to rapid skin–core solidification, non-uniform structure development, and increased internal defects. These effects restrict further molecular rearrangement and ultimately limit mechanical performance enhancement.

Overall, draw ratio, bath temperature, and H_2_SO_4_ concentration synergistically affect the mechanical properties of regenerated viscose fiber, showing a “first beneficial, then detrimental” trend. For high-DP pulp (DP = 789), long molecular chains and dense entanglements efficiently transfer stress under moderate drawing, while sensitivity to coagulation rate requires careful control of temperature and acid concentration to balance regeneration rate and structural uniformity, fully leveraging entanglement advantages for excellent mechanical properties. The optimized conditions are determined as a 1:1.13 draw ratio, 40 °C, and 8% H_2_SO_4_. Under these conditions, regenerated viscose fibers achieve 2.31 cN/dtex tensile strength and suitable elongation, demonstrating the feasibility of high-performance viscose fibers from recycled high-DP cotton pulp.

### 3.4. Mechanical Properties of Viscose Fibers

**Figure 8:**
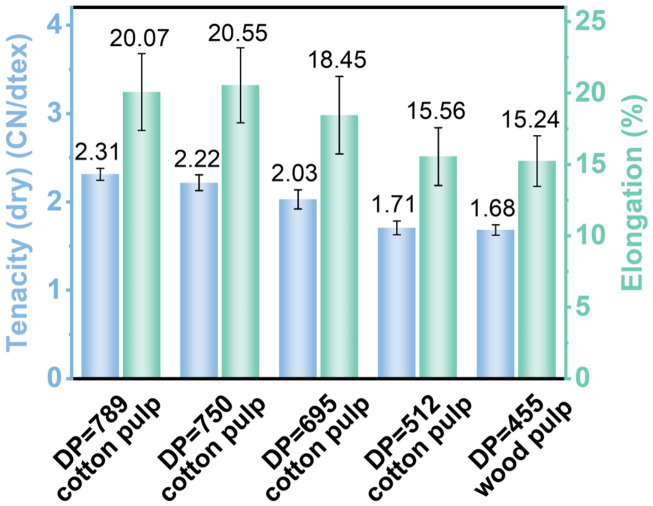
Dry-state tensile strength (blue bars, left axis) and elongation at break (green bars, right axis) of viscose fibers spun from recycled cotton pulps with varying DP and from wood pulp.

Under the optimized spinning conditions, viscose fibers were prepared from recycled cotton pulps of varying DP and commercial wood pulp to systematically investigate the influence of pulp DP on dry-state tensile strength and elongation at break, as shown in Figure 8. Wood pulp-based viscose fibers exhibit a dry-state tensile strength of 1.68 cN/dtex. The dry-state tensile strength of viscose fibers shows a significant positive correlation with the pulp’s DP. Increasing DP from 455 (commercial wood pulp) to 789 (recycled cotton pulp) progressively increases tensile strength from 1.68 cN/dtex to 2.31 cN/dtex. Notably, fiber from DP = 789 pulp exhibits a 37.5% strength improvement compared with commercial wood pulp, highlighting the advantage of high-DP recycled cotton pulp in mechanical performance. It should be noted that the commercial wood pulp used in this study has a DP of 455, which is located at the lower end of the typical DP range for commercial dissolving wood pulps used in viscose production. Therefore, the dry-state tensile strength of viscose fibers prepared from wood pulp with DP = 400–650 reported in the literature (1.5–2.0 cN/dtex) was used for a comprehensive comparison [33,45,46]. Elongation at break demonstrates a similar trend, with viscose fibers from DP = 750 pulp reaching 20.55%, which is significantly higher than the 15.24% observed for DP = 455 wood pulp. Simultaneously, viscose fibers produced from DP = 789 cotton pulp reach 20.07% in elongation at break and also exhibit superior performance compared with wood pulp. Overall, regenerated viscose fibers prepared from recycled cotton pulps, particularly those with DP > 700, exhibit significant mechanical performance advantages over both the commercial wood pulp-based viscose fibers used in this study (DP = 455) and wood pulp-based viscose fibers reported in the literature.

To further assess the statistical significance of differences in the mechanical properties of viscose fibers prepared from pulps with different DP, one-way ANOVA followed by Tukey’s post hoc test is performed at a significance level of *p* < 0.05. ANOVA results show that pulp DP has an extremely significant effect on dry tensile tenacity (*p* < 0.001) and a significant effect on elongation at break (*p* < 0.05). As summarized in Table 2, Tukey’s test indicates no significant differences within viscose fibers from low-DP (455, 512) or high-DP (750, 789) groups for dry tensile tenacity, while fibers from low-DP pulp still have lower tenacity than those from medium- and high-DP pulp. For elongation at break, only partial differences are observed, attributed to the inherent heterogeneity of the viscose fibers. Overall, the statistical analysis confirms that the dry tensile strength of viscose fibers presents a clear increasing trend with pulp DP. This trend can be attributed to the regulation of the molecular entanglement network by pulp DP. Higher DP corresponds to longer molecular chains, resulting in denser and more uniformly distributed entanglements during spinning. This stable entanglement network efficiently transmits stress, promotes axial chain orientation, and enhances dry-state tensile strength. Simultaneously, a moderately dense network allows sufficient deformation, achieving synergistic optimization of strength and toughness. The slightly lower elongation of viscose fibers from DP = 789 recycled cotton pulp compared with DP = 750 likely results from excessive entanglement density, which restricts relative chain slippage. However, the overall performance of regenerated viscose fibers from DP = 789 recycled cotton pulp still surpasses that of commercial wood pulp.

In summary, under optimized spinning conditions, regenerated viscose fibers from recycled cotton pulps exhibit superior dry-state tensile strength and elongation compared with wood pulp fibers. These results confirm that recycled cotton pulps not only possess good spinnability but also offer significant performance advantages, providing support for high-value fiber-to-fiber recycling of waste cotton textiles.

### 3.5. Morphological Structure of Viscose Fibers

**Figure 9:**
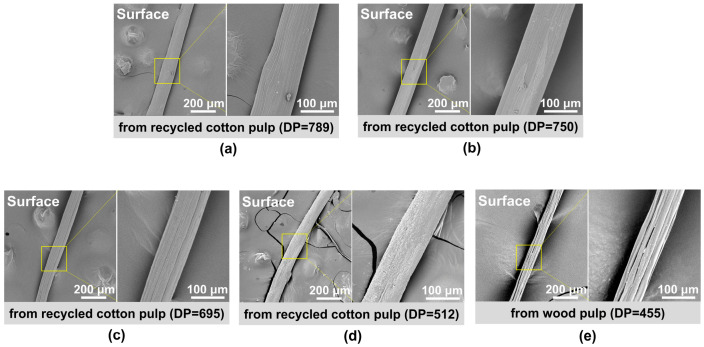
SEM images of the surface morphology of viscose fibers prepared from recycled cotton pulps with different DP (**a**–**d**) and commercial wood pulp (**e**).

The microstructure of viscose fibers from recycled cotton pulps was characterized and compared with wood pulp-based viscose fibers. SEM images of viscose fiber surfaces and cross-sections are presented in Figure 9. The fiber surfaces are generally uniform, showing no apparent fractures or defects. At 800× magnification, grooves aligned along the fiber axis are observed. These grooves represent a typical feature of viscose fibers, resulting from the sequential coagulation of the fiber surface and core, which causes differential shrinkage and axial groove formation. Compared with wood pulp fibers, recycled cotton pulp fibers exhibit smoother, more uniform surfaces with finer groove structures, indicating more stable flow and coagulation behavior during spinning, consistent with the favorable rheological properties observed in Section 3.2.

**Figure 10:**
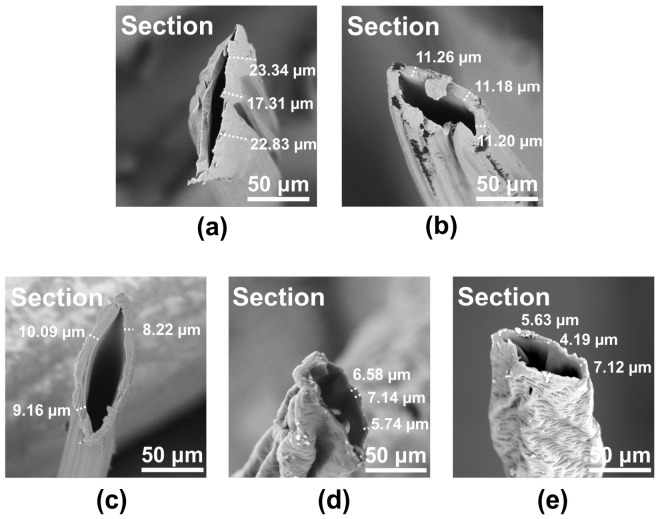
SEM images of the cross-sectional morphology of viscose fibers prepared from recycled cotton pulps with DP values of 789 (**a**), 750 (**b**), 695 (**c**), and 512 (**d**), and from commercial wood pulp (**e**).

Cross-sectional SEM images reveal typical hollow structures in all samples, as shown in Figure 10. This characteristic hollow morphology of viscose fibers originates from rapid surface coagulation during wet spinning, where faster skin formation than core solvent exchange leads to differential shrinkage and a hollow structure. SEM analysis indicates that the viscose fiber’s wall thickness decreases with decreasing pulp DP. Viscose fibers prepared from high-DP recycled cotton pulps (DP = 789) exhibit significantly thicker walls (17.31–23.34 μm), whereas viscose fibers from low-DP recycled cotton pulp (DP = 512) show wall thickness comparable to that of commercial wood pulp fibers (5.63–7.14 μm). Although the measurements may be affected by sample preparation and point selection during image analysis, the overall trend remains clear. In addition, the wall thickness and outer diameter of the obtained viscose fibers differ to some extent from those of industrial viscose fibers, which is mainly attributed to differences between the laboratory-scale spinning setup and the industrial spinning device. Notably, viscose fibers derived from higher-DP recycled cotton pulp generally exhibit thicker and more compact edge walls compared with those from commercial wood pulp. This observation is consistent with the mechanical results discussed in Section 3.4, where regenerated viscose fibers from recycled cotton pulp show higher tensile strength and improved overall mechanical performance.

In conclusion, viscose fibers from recycled cotton pulp maintain typical structural features while exhibiting smoother surfaces and thicker edge walls. These microstructural advantages provide a foundation for producing high-performance regenerated viscose fiber.

## 4. Conclusions

This study systematically investigates the spinning process of recycled cotton pulps with different DPs (512–789). No obvious insoluble content is observed in spinning solutions prepared from our recycled cotton pulps. Furthermore, both the shear-thinning and viscoelastic transition behavior of the spinning solutions are comparable to that of commercial wood pulp, demonstrating excellent spinnability. With increasing DP, apparent viscosity and high-frequency storage modulus increase, while the critical angular frequency shifts to lower values, indicating the formation of a more stable entanglement network that enhances molecular orientation and stress transfer during drawing. Under optimized spinning conditions (draw ratio 1:1.13, coagulation bath temperature 40 °C, sulfuric acid concentration 8%), the viscose fibers obtained from DP = 789 recycled cotton pulp achieve 2.31 cN/dtex dry-state tensile strength and 20.07% elongation at break, exceeding that of wood pulp-based viscose fibers, with smoother surfaces and thicker edge walls. Overall, recycled cotton pulps demonstrate both good spinnability and significant performance advantages in the preparation of regenerated viscose fibers, providing a viable pathway for high-value recycling of waste cotton textiles.

## Figures and Tables

**Table 1:** Carreau–Yasuda rheological parameters of spinning solutions prepared from recycled cotton pulps with varying DP and from commercial wood pulp.

Raw Material	Zero-Shear Viscosity *η*_0_ (Pa·s)	Characteristic Relaxation Time *λ*(s)	Power-Law Index *n*	Goodness of Fit R^2^
Recycled cotton pulp (DP = 789)	94.85	0.68	0.31	0.999
Recycled cotton pulp (DP = 750)	77.85	0.71	0.30	0.999
Recycled cotton pulp (DP = 695)	36.95	0.59	0.29	0.999
Recycled cotton pulp (DP = 512)	9.85	0.42	0.28	0.999
Commercial wood pulp (DP = 455)	9.82	0.41	0.28	0.999

**Table 2:** Significant grouping of the mechanical properties of viscose fibers from different DP pulps based on Tukey’s post hoc test.

Raw Material	Dry Tensile Strength of Viscose Fibers	Elongation at Break of Viscose Fibers
Recycled cotton pulp (DP = 789)	a	ab
Recycled cotton pulp (DP = 750)	a	a
Recycled cotton pulp (DP = 695)	b	ab
Recycled cotton pulp (DP = 512)	c	b
Commercial wood pulp (DP = 455)	c	b

Note: Values in the same column followed by different lowercase letters (a, b, c) are significantly different at *p* < 0.05.

## Data Availability

The data presented in this study are available on request from the corresponding authors.
